# Laser-assisted Rapid Mineralization of Human Tooth Enamel

**DOI:** 10.1038/s41598-017-10082-x

**Published:** 2017-08-29

**Authors:** Muyang Sun, Nier Wu, Haifeng Chen

**Affiliations:** Department of Biomedical Engineering, College of Engineering, Peking University, Beijing, 100871 China

## Abstract

The human body has difficulty repairing damaged dental enamel, an acellular hard tissue. Researchers have sought feasible biomimicry strategies to repair enamel defects; however, few have been successfully translated to clinical applications. In this study, we propose a new method for achieving rapid enamel mineralization under a near-physiological environment. Through treatment with a laser and chelating agents, 15 μm crystals could be grown compactly on an enamel substrate in less than 20 min. The compact crystal layer had similar structure as native enamel prisms and high elastic modulus. This layer also had the potential for further remineralization in saliva. The benefit of using laser can not only speed up the mineralization, but also control the crystal growth precisely where in need. A mechanism for how laser and chelating agents synergistically function is also proposed. This strategy offers a possibility for enamel-biomimicking repair in dental clinics.

## Introduction

Dental enamel is the outmost layer of the human tooth and a highly mineralized tissue in the human body, more than 95% (by volume) of which is composed of carbonated hydroxyapatite^1^. Nanorod-like hydroxyapatite (HAP) crystals are arranged into highly ordered units called enamel prism. Enamel prisms are approximately 5 μm in cross section, are grown along the c-axis, and are approximately 1–2 mm in length. The micro- to nanoscale multi-hierarchical structures of dental enamel provide the remarkable mechanical strength for chewing food and protecting pulp nerves from external stimuli. During the development of dental enamel, ameloblasts play a vital role by producing and secreting the enamel organic matrix, including amelogenin and ameloblastin, which are then calcified with hydroxyapatite to form the enamel. However, upon enamel maturation, ameloblasts will generally polarize, transform into the enamel cuticle, and be removed via mastication. Therefore, mature dental enamel has no living cells to support its regeneration, which means that any enamel damage is permanent.

Many artificial biomimicry strategies have been attempted to repair enamel defects. Different peptides have been used to increase nucleation sites on the enamel surface, such as amelogenin peptide^2^ polyamidoamine (PAMAM)^3^ and 3DSS peptide^4^. These peptides can also function as a mineralization template for HAP formation. Despite the good remineralization results, these methods inevitably have the problem of being time consuming. The HAP synthesis using the above methods usually requires several weeks. Many chemical methods have also been used to achieve rapid crystal growth. Although those methods, such as the hydrothermal reaction^5^ and electrolytic deposition^6^, can achieve mineralization in several hours, the stringent conditions of those chemical reactions limits their clinical application.

In our previous work, we showed the effectiveness of utilizing a chelating agent to chemically regenerate a structure that mimics human enamel under near physiological conditions^7^. The chelating agent could largely retard the nucleation process and promote the growth of crystals longer than 10 μm. Fluoride ions were also added to the mineralization solution, which entered the HAP lattice and replaced OH^−^, forming fluorapatite (FA) crystals with a hexagonal structure. Additionally, fluorapatite has been confirmed to have superior acid resistance^8^. Although the entire operation could be performed in a physiological environment, the process still required several days, which limited the further clinical application.

Although lasers have been used widely for the dental excision of soft tissue and teeth whitening^9, 10^, they have not been used to repair enamel. The photothermal effect of lasers can help heat the local region, which may turn the reaction vessel into a hydrothermal oven. In *et al*. synthesized a ZnO nanowire on a quartz glass wafer^11^. In view of this, we developed a new method to grow FA rapidly on an enamel substrate. A film of compacted, ordered FA crystals was grown via a laser, and this film had a similar structure to human enamel. Scanning electron microscopy (SEM), X-ray diffraction (XRD), X-ray photoelectron spectroscopy (XPS) and nanoindentation were used to evaluate the regenerated layer. To further explore this mechanism, Fourier transform infrared spectroscopy (FT-IR) and TEM analysis were used.

## Methods

### Sample and mineralization solution preparation

Non-carious human teeth were obtained from Peking University School of Stomatology under an agreement with the patients by signing an informed consent. The protocol for processing human tissue specimens was reviewed and approved by the University’s committee on Use and Care of Human Tissue Specimens. The methods were operated in accordance with the Declaration of Helsinki (2008). The whole tooth was cut into regular shapes, ensuring the surface was sufficiently flat. To minimize the individual difference from different teeth, each tooth was divided into 4–6 parts, one of which was used as the control sample. Each series of experiments was finished on the same tooth. After the cutting, the tooth bulks were ultrasonically cleaned for 30 s and smeared with a thin film of graphite. Cylinder spectrographic graphite electrodes (Sinosteel Corporation, China) were used as our graphite source. The process of smearing was just like drawing with a pencil on the paper. Before smearing, tooth surface was wiped dry. To prepare the mineralization solution, HEDTA and CaCl_2_ were mixed together in DI water. Due to the low solubility of HEDTA in water, the pH of the mixture was adjusted to 6, which helped HEDTA dissolve. After all the Ca^2+^ and HEDTA was dissolved, NaH_2_PO_4_·2H_2_O was dissolved in DI water and poured in the mixture above. Then, the pH was adjusted to 6.0, and NaF was added to the solution. The final suspension contained N-(2-hydroxyethyl) ethylenediamine-N,N′,N′-triacetic acid (HEDTA), CaCl_2_, NaH_2_PO_4_ and NaF, with the molar ratio of Ca^2+^, PO_4_^3−^ and F^−^ maintained at 5:3:1 and the Ca^2+^ concentration remaining greater than 0.1 M. The concentration of HEDTA was 10–20% higher than that of Ca^2+^ for long-term storage. The pH of the precursor solution was adjusted to 6.0 using NaOH and HCl.

### Laser-assisted crystal growth

**Figure 1:**
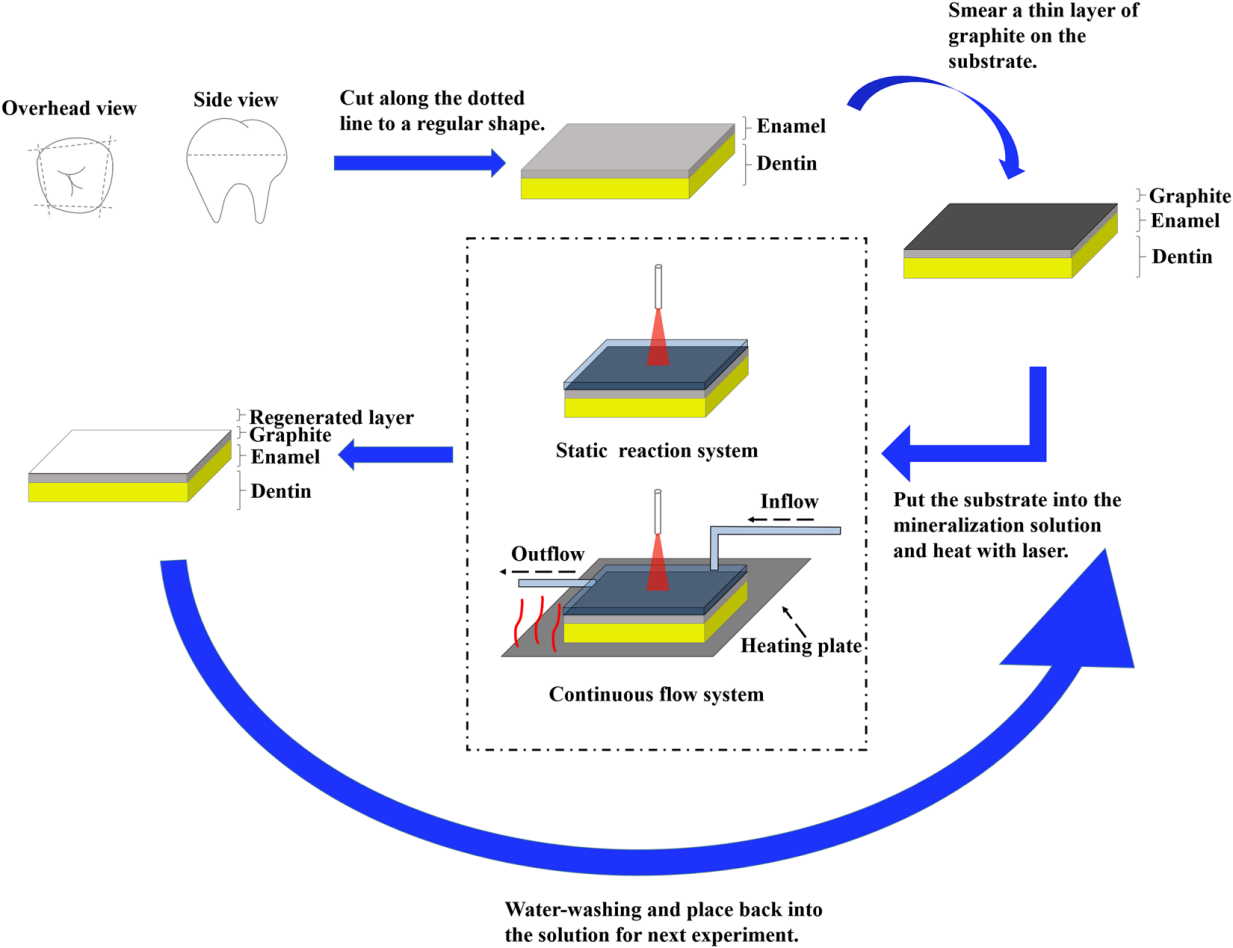
Schematic illustration of the laser-assisted mineralization process. Fluorapatite is grown on the surface of enamel. To facilitate the absorption of the laser light, graphite was smeared on the surface. After preparing the sample, we synthesized a special mineralization precursor solution and immersed the sample in that solution. The laser treatment included two different system. The static reaction system was the primary model and applied for all the characterizations in this work, while the continuous flow system was the improved model. After laser treatment, the sample was cleaned and retreated again with a new batch of the solution.

A mini dental diode laser system with a wavelength of 980 nm (Wuhan Gigaa Optonics Technology Co., China) was used as our laser source. The whole substrate was immersed in a precursor solution, with the fluid maintained at a level 1–2 mm higher than the substrate. A laser beam (continuous, 980 nm) with a spot size of 0.4 mm was focused vertically onto the substrate (Fig. 1). Because of the high transmissivity of the diode laser in water and hydroxyapatite^12^, there was a risk that the laser process could induce potential thermal side effects. Therefore, based on a series of trials (Fig. S1), we chose 2 W as the laser power, and a single processing time was 3 min, ensuring that the pulp temperature increased by less than 6 °C (Laser group)^13^. The laser light used here was not parallel light; thus, the distance from the laser fiber to the enamel was dependent on the energy density, which was estimated to be 0.64 W/mm^2^. After the laser process, the substrate was rinsed with clean water or received another ultrasonic treatment. All samples were air dried for further analysis. The laser-treated samples were then immersed for 3 days in artificial saliva^14^, which contained 50 mmol/L 4-(2-hydroxyethyl)-1- piperazineethanesulfonic acid (HEPES) buffer, 1.14 mmol/L calcium chloride, 0.59 mmol/L potassium dihydrogen phosphate, and 30 mmol/L potassium chloride, adjusted to pH 7.0 using potassium hydroxide.

### Detection of the pulp temperature change

Teeth employed here were human third molars. Teeth were sectioned 5 mm below the level of the amelocemental junction and drilled along the pulp chamber, ensuring that the ZNHW-II thermocouple used for temperature detection and the control system (Yuhua Instrument, China) could be sealed at the end of the chamber using wax. The tooth surface was smeared with a thin layer of graphite. The whole tooth and thermocouple were fixed on the bottom of the beaker and immersed in water. The specific parameters, such as the depth of water and the distance between the laser fiber and teeth, were the same as for the above experiments. The power outputs ranged between 1 W and 3 W. Each round of the experiment lasted 5 min with a 20 min cool down. Every parallel test contained 3 samples, and the results are presented as the mean ± standard deviation.

### Characteristics of the mineralized crystal

The morphology of the reconstructed layer was observed on a scanning electron microscope (FEI company, NovaSEM 430, USA) operated at 10–20 kV. For higher resolution in the SEM image, all samples were coated with an Au/Pd film. The crystalline phase of the newly grown layer was examined by X-ray diffraction (Rigatu, D/max, USA) with Cu Kα radiation (λ = 1.5405) at 40 kV and 100 mA. An X-Ray Imaging Photoelectron Spectrometer (Kratos, Axis Ultra, UK) was used to analyze the chemical composition of the regenerated layer, with Al Kα X-rays at 40 kV with a power of 225 W.

A TriboIndenter (Hysitron Inc., US) with a 100 nm Berkovich tip at a maximum load of 1000 μN was used to measure the elastic modulus and hardness of our samples. All specimens were cut thinly so that the weak strength of dentin would not affect the final result. Three indents were performed for each sample, and results were analyzed statistically.

### Evaluation of the effects of HEDTA and temperature on the crystal growth

Infrared spectroscopy was conducted using a Magna-IR 750 Fourier transform infrared spectrometer (Nicolet, USA) and the KBr pellet method. The scan range was from 400 cm^−1^ to 4000 cm^−1^, and the resolution was 4 cm^−1^. Powder X-ray diffraction patterns were obtained using an XRD-Terra (INNOV-X, USA) with Cu Kα radiation (λ = 1.5405) at 30 kV and 330 μA. A Tecnai F30 high resolution electron microscope (FEI, USA) operating at 300 kV was used for normal TEM imaging. The sample was pipetted to the holey-carbon film and dried before TEM measurements.

A potentiometric titration experiment was performed in a beaker (200 mL) filled with phosphate buffer (100 mL, 0.1 M NaH_2_PO_4_·2H_2_O and 0.03 M NaF). For experiments using HEDTA, 0.1 g HEDTA was dissolved in the phosphate buffer. The pH of the phosphate buffer was adjusted to 6.00. CaCl_2_ solution (0.25 M) was added dropwise at a rate of 3 mL/h. The pH was stably maintained at 6.00 using sodium hydroxide solution, and the stirring rate remained the same as during the calibration process. The calcium potential was monitored and recorded once per 30 s. The electrodes, beaker and burette tips were cleaned with hydrochloric acid (10%) and carefully rinsed with DI water after every experiment. All other experiments were performed at RT (25 °C), whereas experiments on the temperature effect were performed at 35 °C (electrodes should be recalibrated at 35 °C).

### Simulation of the laser-induced temperature distribution

The simulation of temperature distribution after laser treatment of a simplified tooth model was conducted using a two-dimensional finite difference method. The tooth was represented as a cylinder with uniform isotropic photon absorption properties and thermal properties. Light scattering was not considered. The boundary conditions of the tooth were forced convection with stirred water. Due to the symmetric configuration, the discretized equations were simplified and solved using a two-dimensional finite difference method with a central difference scheme. Both graphite-smeared and untreated samples were simulated.

### Crystal growth on different substrates

To grow crystals on polystyrene plates and glass sheets, we smeared graphite on the substrates to facilitate laser absorption. The laser used here was a 980 nm diode laser (Hi-Tech Optoelectronics Co., LOS-BLD-0980-002W-C/P, China) with a spot size of 0.1 mm. For fluorapatite, the final mineralization solution contained 0.1 M CaCl_2_, 0.1 M HEDTA, 0.06 M NaH_2_PO_4_ and 0.02 M NaF, pH 6.00. For calcium carbonate, the final solution contained 0.15 M CaCl_2_, 0.1 M HEDTA and 0.15 M Na_2_CO_3_, pH 6.00. For zinc oxide, the final solution contained 0.2 M ZnSO_4_·7H_2_O and 0.1 M HEDTA, pH 6.00. The laser process lasted 4 min.

### Rapid mineralization of enamel via continuous flow system

As shown schematically in Fig. 1, an ALC-IP900 injection pump (ALCBIO, China) was used. The injecting rate was set 0.1 mL/min. Mineralization solution containing 0.6 M CaCl_2_, 0.36 M NaH_2_PO_4_, 0.12 NaF and 0.78 M HEDTA was prepared. A C-MAG HS 4 heating magnetic stirrer (IKA, Germany) was used as the heating plate, the temperature of which was set 40 °C. Graphite-coating enamel pieces were placed on the heating plate, immersing in the mineralization solution. Diode laser (2 w) was vertically irradiated on the substrate for 3 minutes. During the treatment, the pump kept injecting fresh mineralization solution into the vessel. Each sample was treated 5 times and then washed by DI water.

## Results

### Characteristics of the regenerated enamel

**Figure 2:**
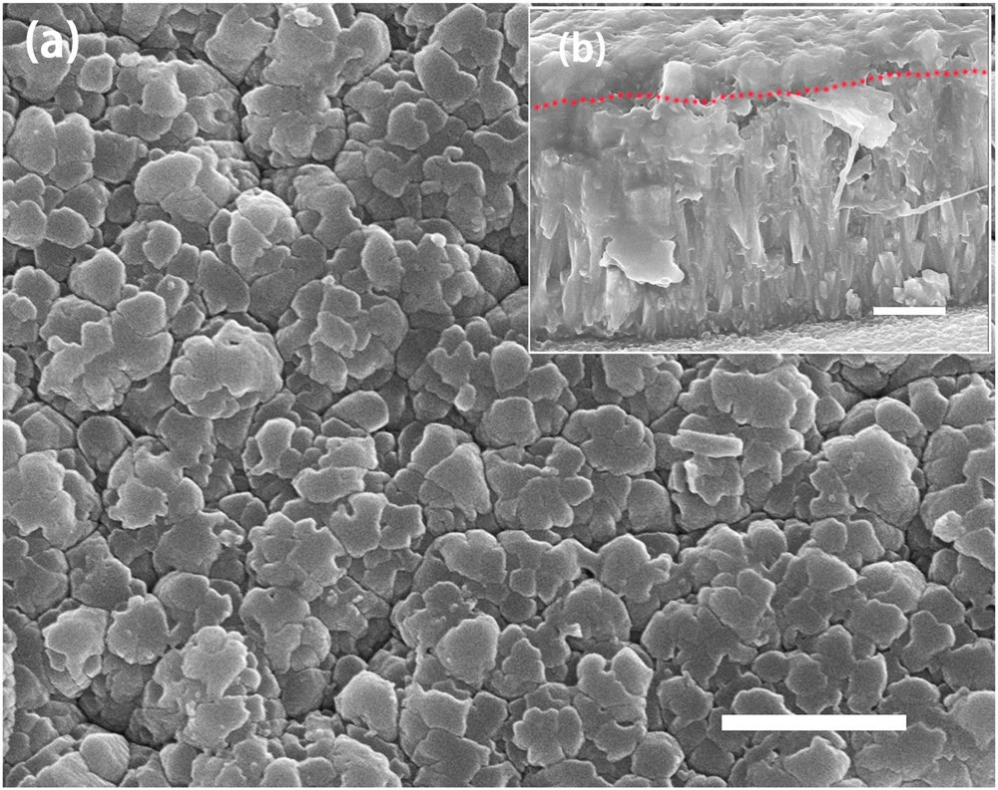
(**a**) SEM image of a regenerated film that was treated 5 times via laser and then immersed in the artificial saliva for 3 days; (**b**) Cross-sectional image of the sample (**a**). The dotted red line in (**b**) shows the boundary between the newly grown layer and the previously grown layer. Scale bars, 1 μm.

**Figure 3:**
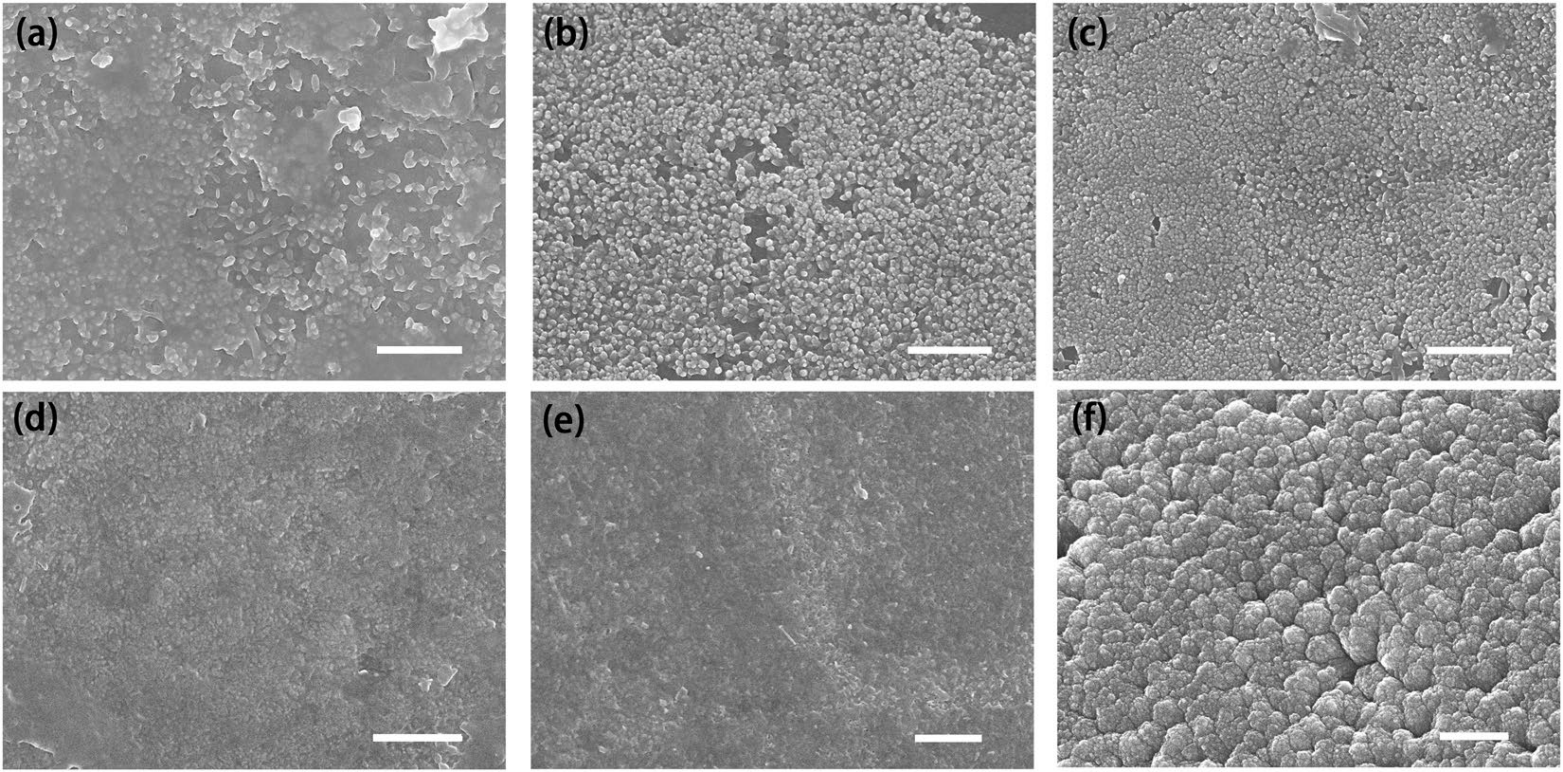
SEM images of our results. (**a**–**d**) Images of laser-treated samples under different Ca^2+^ concentration conditions: (**a**) 0.1 M, (**b**) 0.2 M, (**c**) 0.3 M. (**d**), Tooth without any treatment, the control group. (**e**) Image of the control group immersed in the artificial saliva for 3 days. (**f**) Image of the laser-treated group following 5 treatments. Scale bars, 1 μm.

Figures 2 and 3 show SEM graphs of the enamel surfaces under different processing conditions. Comparison with the natural enamel surface (Fig. 3d) indicated the presence of crystal grains on the substrate for all the remineralized groups. The diameter of the spherical crystals was nearly 50 nm. As the Ca^2+^ concentration and treatment times increased, the apatite particles grew and merged into larger crystals (Fig. 3a–c,f). Additionally, tiny apatite particles aggregated around the surface. After several treatments, the aggregated particles eventually formed a compact crystal film with the thickness more than 1 μm along the c-axis. At the top side of the layer, a thin film with disordered apatite particles could also be observed. It seemed that the newly grown crystals would keep growing at a similar rate and were unaffected by the previous treatment (Table S1). After immersion in artificial saliva for 3 days, the surface of the natural enamel had few changes (Fig. 3e), whereas the laser-treated group showed a tendency to form hexagonal crystals (Fig. 2a). The cross-sectional SEM (Fig. 2b) showed that the regenerated layer had a similar structure as the enamel prism.

**Figure 4:**
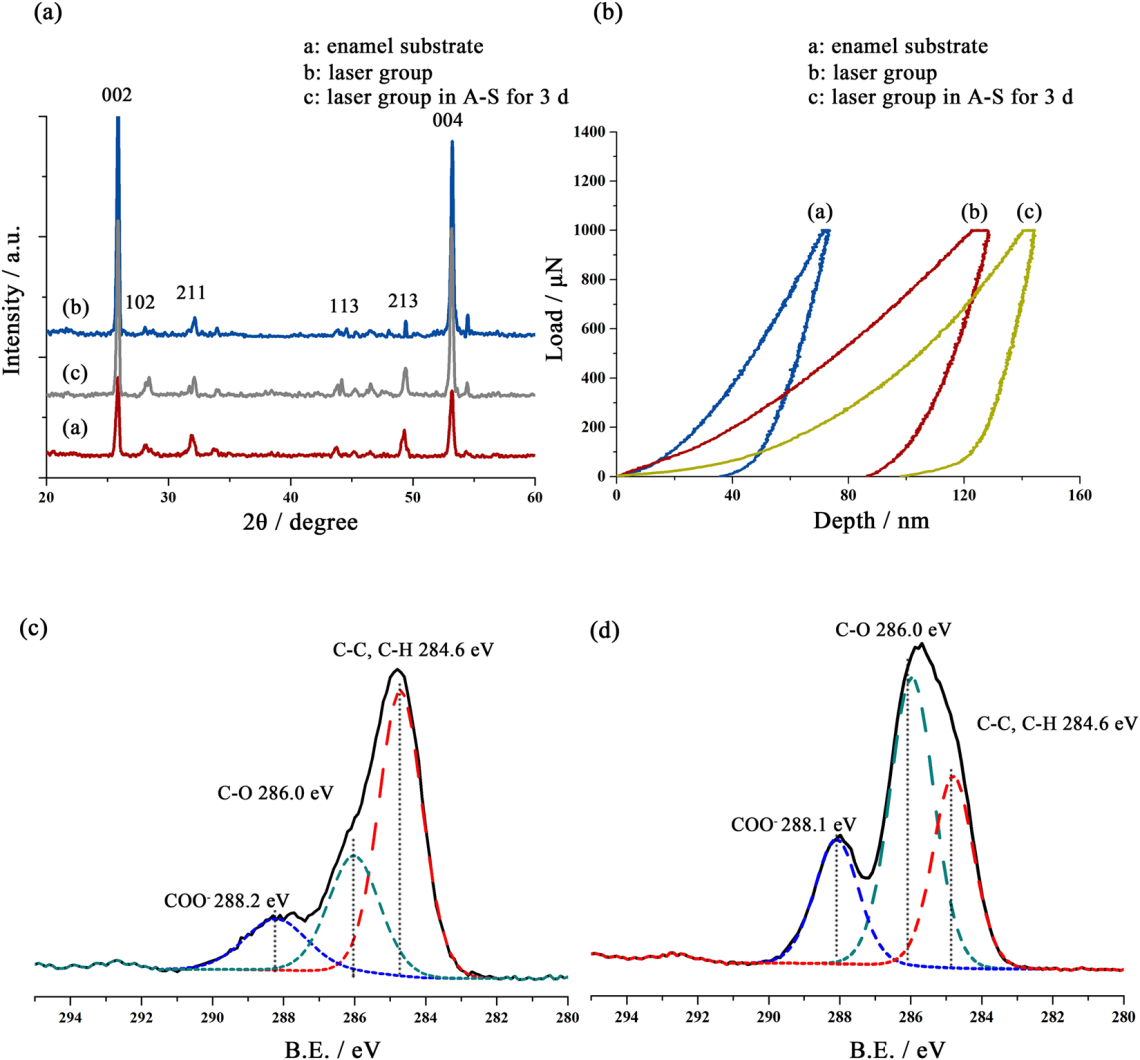
Characteristics of the regenerated layer. (**a**) X-ray diffraction patterns of enamel, laser-treated enamel, and enamel remineralized by laser treatment and A-S for 3d. (**b**) Loading-unloading curves from nano-indentation tests at a maximum loading force of 1000 μN. (**c**,**d**) Detailed XPS spectrum of the C 1 s from the native enamel (**c**) and laser-treated enamel (**d**).

XRD spectra were used to confirm the structure of remineralized crystal. Figure 4a shows that positions of the main peaks of all three samples were in accordance with those from the standard XRD pattern of fluorapatite. By contrast, crystals from the enamel substrate and the newly grown layer had notably sharp and intense 002 and 004 peaks. Herein, the area (A) of the diffraction peaks at 2θ = 25.8° for (002) and 49.2° for (213) were used for a semi-quantitative evaluation of the orientation of crystals. To minimize any systematic error resulting from using different samples, all samples used were from the same tooth. The enamel substrate had a typical ratio (Q) of A_002_:A_213_ = 2.11, which was attributed to enamel prisms. After laser-assisted crystal growth, Q was 25.3, which was ten times higher than that of the substrate. This difference matched the SEM result, whereby the tiny crystals in the laser groups were closely packed and were grown perpendicularly against the surface (Fig. 2b). The mineralization process in artificial saliva reduced the Q to 4.46, indicating further maturation of the fluorapatite crystal.

The mechanical properties of the regenerated enamel layer were evaluated using nano-indentation. The loading-unloading curves are provided in Fig. 4b. Detailed elastic modulus and hardness values are listed in Table S1. Compared with the natural enamel substrate, the remineralized layer had obviously lower values for both the elastic modulus and hardness. After 3 days of mineralization in artificial saliva, the elastic modulus rebounded to 60.54 ± 0.83 GPa. Although the thickness of the regenerated layer was several microns, the rapid growth led to the formation of a calcium-deficient crystal. Calcium ions in the artificial saliva gradually entered the poor-crystalline apatite lattice and matured the crystal. However, the hardness changed little when the mineralized teeth were treated by artificial saliva, which may result from the change in crystal orientation.

The detailed XPS spectrum of the C 1 s region of the natural enamel surface showed a primary C-C, C-H peak at 284.6 eV and a weak C-O peak at 286.0 eV (Fig. 4c)^15^. A much weaker COO^−^ peak at 288.2 eV was also observed. The complete C 1 s peak was attributed to the specific composition of enamel and a surplus of common carbon contamination. However, the narrow-scan spectrum of the enamel treated with mineralization solution showed an obvious increase in the intensity of the C-O, C-H peak and the COO^−^ peak, which resulted from the carboxyl group of N-(2-hydroxyethyl)ethylenediamine-N,N/,N/-triacetic acid (HEDTA) (Fig. 4d). The shift in the COO^−^ peak was due to the chelating effect between COO^−^ and Ca^2+^ ^16^.

### Mechanism analysis on the mineralization model

**Figure 5:**
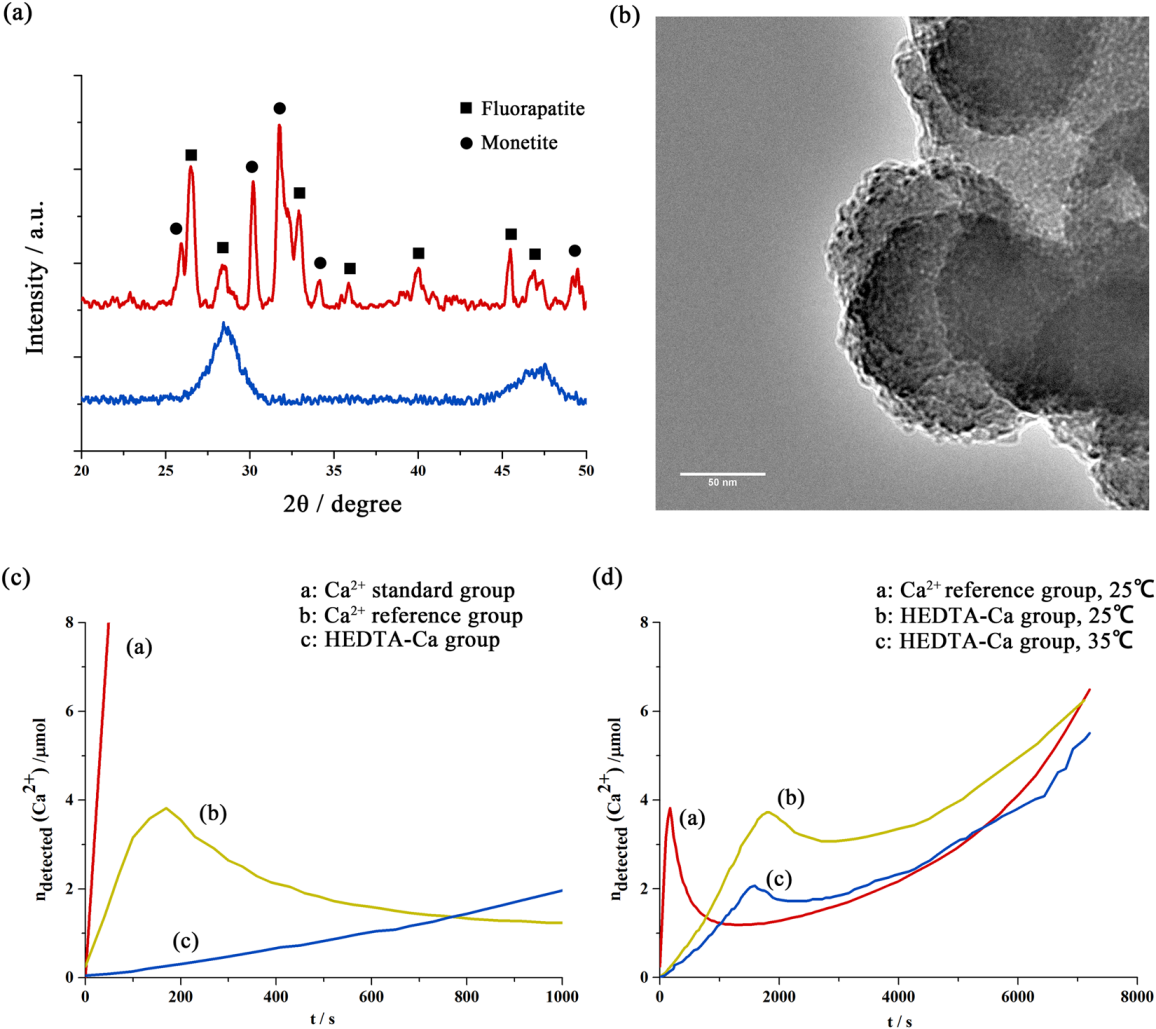
Effects of HEDTA and temperature on the nucleation and growth of fluorapatite (**a**) shown in the XRD patterns of precipitates from the HEDTA solution (blue line) and the non-HEDTA group (yellow line). (**b**) TEM of the precipitate from the HEDTA solution, which showed PNCs of approximately 5 nm aggregating around ACP. (**c**,**d**) Time course of the amount of free calcium ions measured by a calcium ion-selective electrode (Ca-ISE) in different phosphate solutions at different temperatures.

To study the effect of chelating agents in these experiments, FT-IR, XRD and TEM were used to analyze the structure and composition of the mineralization solution. When the Ca^2+^ solution was mixed with the PO_4_^3−^ and F^−^ solution at pH 6, the mixture became turbid, and large amounts of apatite particles precipitated immediately. After centrifugation and lyophilization, the supernatant and precipitate were analyzed, respectively. The XRD result shown in Fig. 5a indicates that the precipitate was composed of fluorapatite and monetite, consistent with the FT-IR results. FT-IR of the supernatant from the non-HEDTA solution showed few peaks between 1100 cm^−1^ and 1000 cm^−1^, indicating that nearly all phosphate groups had been transferred into crystals (Fig. S6a). By contrast, in the HEDTA-Ca group, there was much less precipitate. The XRD result from the precipitate of the HEDTA-Ca group showed a strong and broad peak at approximately 2θ = 30°, which is a characteristic of amorphous calcium phosphate (ACP). By analyzing the supernatant of the HEDTA group via FT-IR (Fig. S6b), we also confirmed the existence of pre-nucleation clusters (PNCs) in the supernatant of the HEDTA-Ca group, consistent with the TEM results (spherical particles ~5 nm, Fig. 5b). A detailed discussion about FT-IR and second-derivative spectrum is provided in the Supplementary Information.

To further elucidate the effect of chelating agents on retarding nucleation, mineralization experiments were performed by adding calcium solution at a constant rate into an excess phosphate and fluoride solution^17^ (Fig. 5c). The increase in the amount of free calcium ions in both phosphate groups was flattened compared to that in the standard group (only contained Ca^2+^) due to the formation of Ca-P clusters. The much flatter increasing curve of the HEDTA-Ca group was attributed to the chelating effect between the Ca^2+^ and carboxyl groups. After 170 s, nucleation occurred in the non-HEDTA group, which was 25 min faster than the nucleation in the HEDTA-Ca group (Fig. 5c). The slow nucleation process in the HEDTA-Ca group demonstrated the efficiency of HEDTA for inhibiting the nucleation. When the nucleation occurred, nearly 3.8 μmol of free Ca^2+^ existed in the HEDTA-Ca group, which was much higher than the theoretical amount of free calcium ions chelated by HEDTA (pK_eq_ = 8.6) in DI water (~0.1 μmol). Therefore, only a portion of the HEDTA molecules participated in the binding to free calcium ions, and others stabilized pre-nucleation clusters. As nucleation began, the concentration of unbound Ca^2+^ ions dropped immediately. Along with the continuously added calcium solution, most of the PNCs and free Ca^2+^ ions continued nucleating and were incorporated into the lattice of ACP, leading to a slow increase in the rate of unbound Ca^2+^.

The effect of temperature was also evaluated using the above experiments (Fig. 5d). When the temperature of the reaction was raised to 35 °C, an additional 40% of the free Ca^2+^ ions in the HEDTA-Ca group formed clusters. Furthermore, the nucleation time was 10% faster than at 25 °C. As the temperature increased, the amount of free Ca^2+^ ions required for nucleation decreased by half. This phenomenon also appeared in the Ca standard group, where the required amount of free Ca^2+^ ions decreased to 20%. The temperature increase was attributed to a much faster rate for nucleation. The temperature increase also prolonged the platform stage, which led to continuous crystal growth.

**Figure 6:**
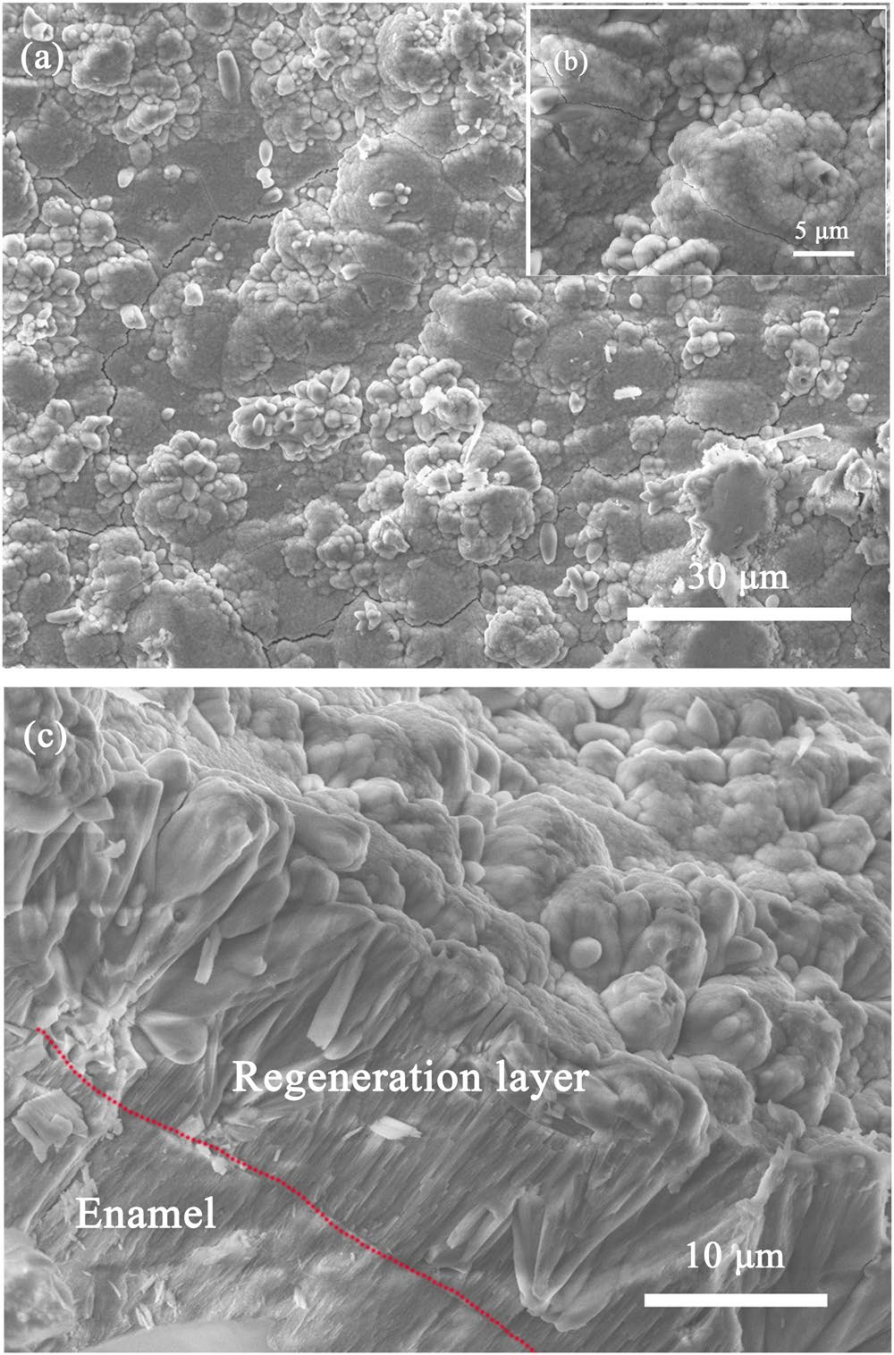
SEM images of rapid mineralization of enamel via continuous flow system and the schematic illustration (**a**,**b**) images of crystal film surface with different scale bar; (**c**) cross-section image of the sample (**a**).

Based on experiments above, we moved further to develop a system of rapid mineralization of enamel. A continuous flow system was designed and the reaction temperature was also raised from room temperature (25 °C) up to 37 °C. The flow system kept injecting fresh mineralization solution into our reaction vessel. The injecting speed was screened so that it won’t cool down the reaction temperature too much. As shown in Fig. 6, after 5 times treatment (totally 15 minutes), the newly grown crystal layer was about 15 μm. The newly developed enamel layer also appeared to be more compact than before.

## Discussion

In general, the mineralization process can be divided into two stages: crystal nucleation and growth. In traditional methods of mineralization trials, researchers must sacrifice time to achieve a highly pure crystal. For enamel mineralization, however, the time and crystal growth should be balanced. Given the complexity of crystal growth, it is easier to modulate the nucleation rate. Therefore, we turned to another way of achieving crystal growth that maintains continuous nucleation during crystal growth.

In classical nucleation theory, the nucleation rate is usually described by the following term^18, 19^:

1 $${\rm{J}}\propto \exp (-{{\rm{E}}}_{{\rm{A}}}/{{\rm{k}}}_{{\rm{B}}}{\rm{T}})\cdot \exp (-{{\rm{\Delta }}{\rm{G}}}_{{\rm{ex}}}/{{\rm{k}}}_{{\rm{B}}}{\rm{T}})$$

The first exponential term is related to the kinetic barriers with the energy E_A_ (k_B_: Boltzmann constant, T: absolute temperature), which arise from individual reactions, such as rearrangements within the nucleus. The second exponential term refers to the positive excess free energy of the newly formed phase. Additionally, ΔG_ex_ represents for the thermodynamic barrier for nucleation. The free energy of the interface between the mineral, the surrounding solvent and the substrate determine the value of ΔG_ex_. Herein, ΔG_ex_ can be expressed as follows:

2 $${{\rm{\Delta }}{\rm{G}}}_{{\rm{ex}}}={{\rm{B}}{\rm{\alpha }}}^{3}{{\rm{\sigma }}}^{-2}$$

where B is a constant related to the shape and density of the nucleating solid, α is the interfacial free energy, and σ is the supersaturation.

Based on the nucleation function (1), temperature can markedly change the nucleation rate, which has been confirmed by numerous studies. However, although the enamel substrate may have a lower energy barrier to nucleation, bulk heating will cause homogeneous nucleation in solution prior to heterogeneous nucleation on a solid substrate. The homogeneous nucleation will consume nearly all PNCs and ions, limiting further crystal growth (Fig. S6a). In addition, during clinical surgeries, the oral environment will not tolerate high temperature, which may induce dental ulcers. In summary, heating a limited region, especially on the surface of the substrate, would be our preferred choice. A diode laser was chosen as the heat source because of its low absorption by water. Because the main component of enamel, hydroxyapatite, also has low absorption of the diode laser, the substrate was smeared with a thin film of graphite to facilitate absorption. Thus, only the surface of the substrate was heated, and the natural convection occurring in solution could effectively dissipate extra heat.

**Figure 7:**
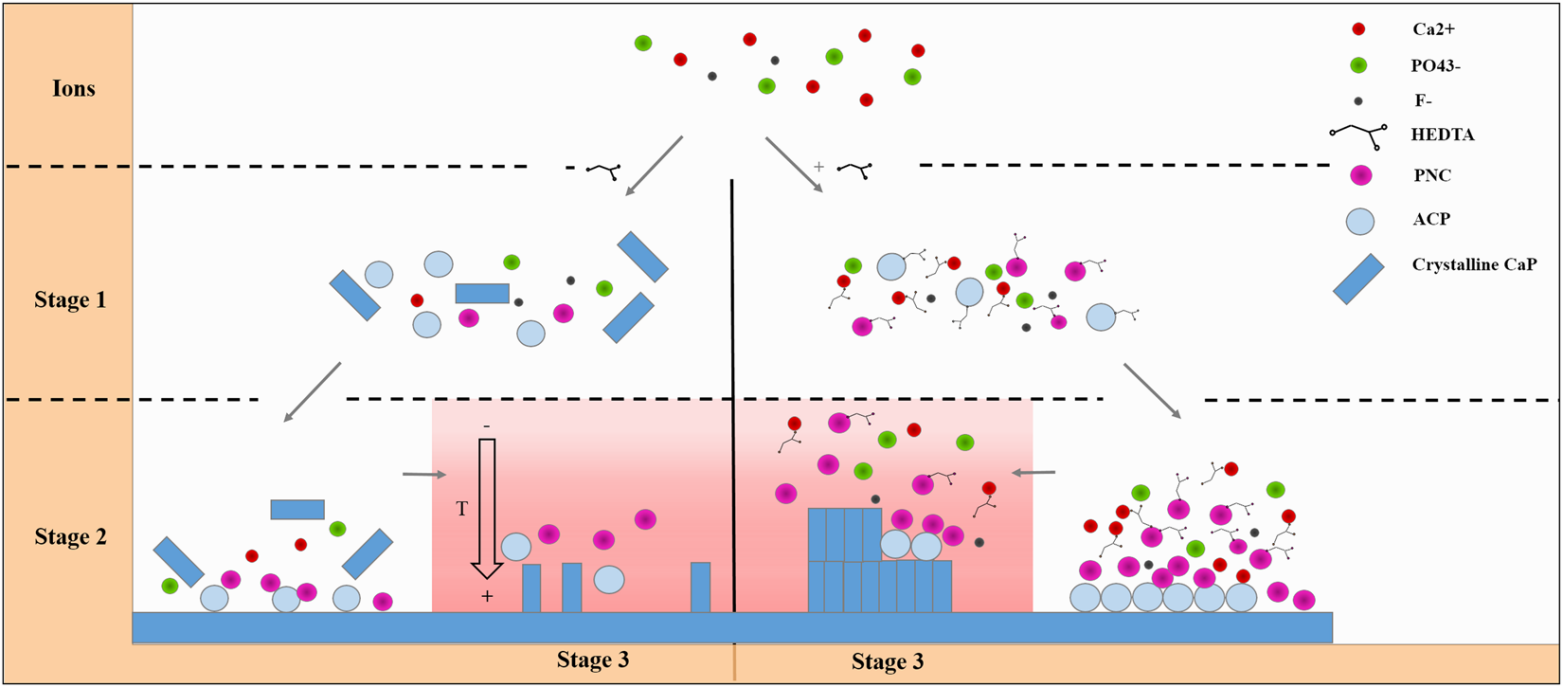
Scheme depicting the possible mechanism of the repair process.

A high concentration of Ca^2+^ can also accelerate nucleation based on equations (1) and (2) via increasing the supersaturation. However, high supersaturation may also lead to over-rapid homogeneous nucleation with few crystals growing on the substrate under that condition^20^. Inhibitors, such as aspartate and glutamate, have been proven to effectively modulate the nucleation process. The carboxyl and phosphate groups of these acidic amino acids can chelate calcium and bind to specific crystal faces, which will retard nucleation^21, 22^. However, few studies have used those agents for mineralization on a solid surface, and the relevant mechanism is unclear. In our previous work, we showed the possibility of using organic chelating agents, such as EDTA and HEDTA, to promote the mineralization process. Combining previous studies^23, 24^ and the above observations, we propose a possible mechanism to elucidate how chelating agents and laser promote rapid mineralization (Fig. 7).

In the mineralization solution, Ca^2+^ and PO_4_^3−^ form PNCs, which have been confirmed as the building unit of an amorphous bulk phase. In the absence of inhibitors, PNCs will spontaneously nucleate and be used up (Fig. 7, stage 1; Fig. S6a), leaving few PNCs and ions for further nucleation and long-term growth (Fig. 7, stage 2). The uncontrollable nucleation will also induce the growth of different types of apatite (Fig. 7a). By contrast, the addition of HEDTA can either chelate Ca^2+^ or bind to the surface of PNCs and ACP. The binding of HEDTA will make those particles negatively charged, thus maintaining the stability of particles through electrostatic repulsion. Although spontaneous nucleation will still occur, only some of the Ca^2+^ will participate in this process, with most being stabilized by inhibitors (Fig. S6b). When the substrate was heated by a diode laser (Fig. 7, stage 3), the heated region will have lower interfacial free energy and a faster nucleation rate. The isolated PNCs will then agglomerate near the substrate and present as small aggregations, with a subsequent transformation into ACP (Fig. 3a–c). During this process, the ACP will recruit pre-nucleation clusters that merge into the crystal and make them mature. Meanwhile, PNCs will continue to nucleate and agglomerate on the surface, forming a new layer composed of unordered ACP (Fig. 2b). Free Ca^2+^ ions will be slowly released from the HEDTA-Ca complex and will form new pre-nucleation clusters, which act as a Ca^2+^ reservoir for long-term growth. HEDTA will chelate apatite on the surface (Fig. 4d); thus, this modified apatite layer will act as a template for further mineralization by artificial saliva (Fig. 2a).

With this laser-assisted strategy, we put forward a continuous flow system for faster enamel mineralization (Fig. 6). Fresh mineralization could keep offer pre-nucleation clusters and ions. The reaction temperature rose up from the top down, recruiting them growing on the latest crystal film. Besides, crystals can grow not only on the enamel substrate but also on a metal surface, a polystyrene plate, and a glass sheet (Fig. S3). Whether smearing graphite on the surface is required for crystal growth depends on the light-absorbing characteristics of the material. In clinical operations, EDTA has been used as detoxification reagents curing lead poisoning and demineralized reagents on teeth, which demonstrated its low toxicity. Considering the structure similarity between EDTA and HEDTA (one carboxyl agent on the EDTA molecule is replaced by hydroxyl group), HEDTA could also have potential application in oral conditions. Therefore, detailed clinical application protocols were in preparation with dentists, and further animal experiments were necessary to fully test the result.

However, considering the high transmittance of the diode laser by hydroxyapatite, the laser source used here could still easily cause overheating of tooth pulp, which would induce the permanent death of pulp nerve cells. A laser with a higher wavelength (>2 μm) was more easily absorbed by teeth; thus, further study is needed to verify the effectiveness of replacing the diode laser. Another problem is related to teeth whitening. Graphite would be embedded in the mineralized layer, causing a light black color in teeth. We tried to use up-conversion fluorescent apatite particles (converting 980 nm to 543 nm and 654 nm) to replace the graphite for deposition on the substrate. The result was unsatisfactory because of the uneven distribution of those particles on the surface.

In summary, the present work shows that we have successfully developed a layer consisting of an ordered and well-compacted fluorapatite crystal film that forms rapidly on the enamel substrate with laser treatment. The film is analogous to the natural enamel in its chemical components and microarchitectural structure. The precise control of the mineralization via a laser can also avoid the formation of dental calculus. In contrast to current regenerative medicine using stem cells, this laser-assisted strategy is simple and safe. This work demonstrates the potential application of laser-assisted rapid mineralization for enamel-biomimicking repair in dental clinics.

## Electronic supplementary material

Supplementary Information
