# Supplementary material for: Laser-assisted Rapid Mineralization of Human Tooth Enamel
Source: Sci Rep. 2017 Aug 29;7:9611. doi: 10.1038/s41598-017-10082-x (PMC5574885; doi:10.1038/s41598-017-10082-x)
Supplement: Supplementary file 1 — Supplementary Information [file 41598_2017_10082_MOESM1_ESM.pdf]

# Supplementary Information

# Laser-assisted Rapid Mineralization of Human Tooth Enamel

Muyang Sun, Nier Wu, Haifeng Chen[1]

1. Department of Biomedical Engineering, College of Engineering, Peking University, Beijing, 100871, China

**Corresponding Author:** Haifeng Chen, Ph.D.

Department of Biomedical Engineering, College of Engineering, Peking University, Beijing, 100871, China

E-mail: haifeng.chen@pku.edu.cn;

Fax: +86-10-62754396;

Tel: +86-10-62754396.

[1] Department of Biomedical Engineering, College of Engineering, Peking University, Beijing 100871, China. E-mail: haifeng.chen@pku.edu.cn; Fax: +86-10-62754396; Tel: +86-10-62754396

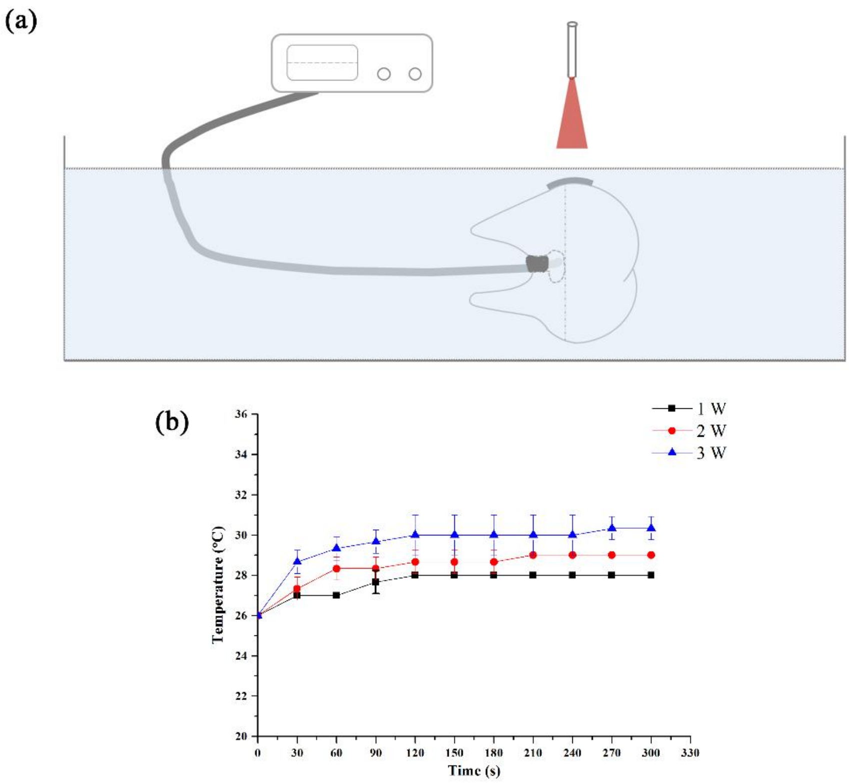


**Figure S1. Scheme of pulp temperature measurements (a) and the temperature change dependent on the power (b)**

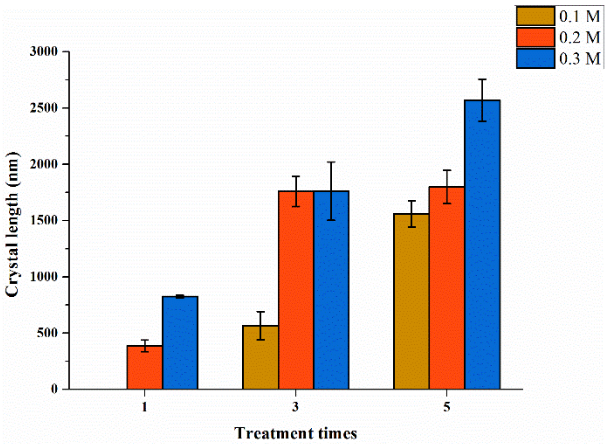


**Figure S2. Crystal length's dependence on treatment times and concentration. Tooth substrates were laser treated under different $Ca^{2+}$ and HEDTA concentrations. After every treatment, the substrate was cleaned by DI water and placed back to a new batch of the remineralization solution. For those groups treated many times, the treated zone was kept as the same by locating with the red visible light.**

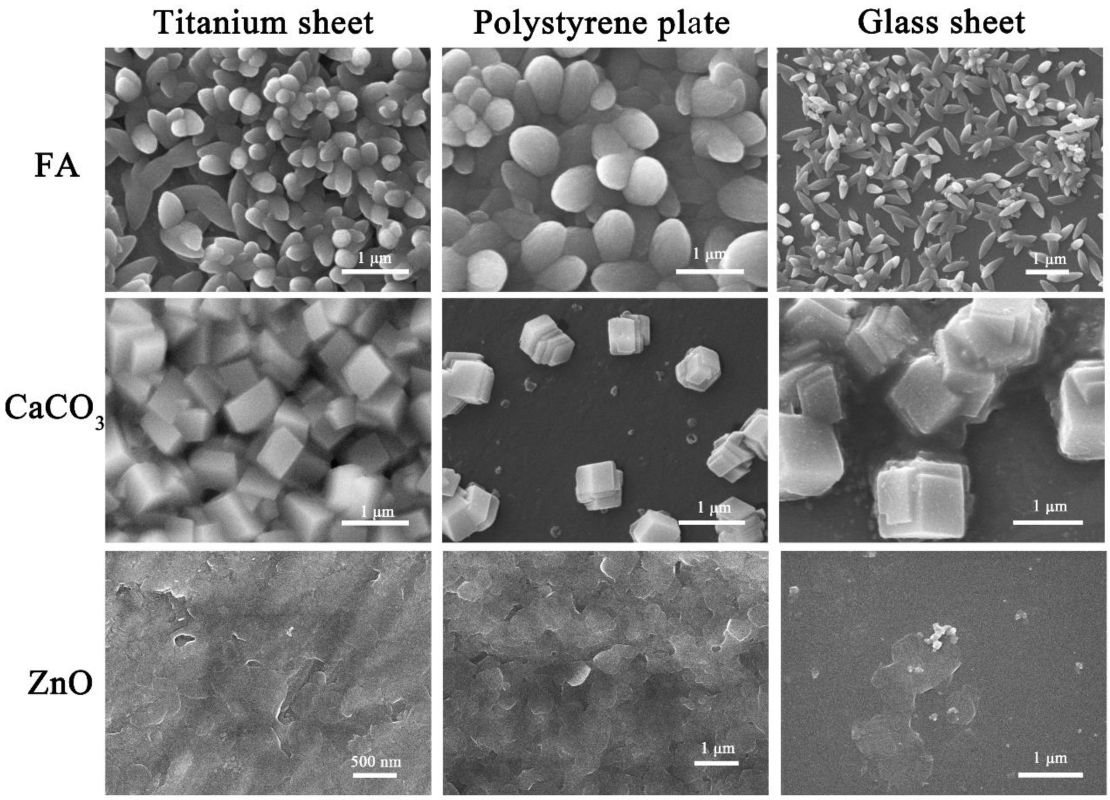


**Figure S3. SEM results of growing different crystals on different substrates.**

**Table 1 Mechanical properties of natural and regenerated enamel by nanoindentation tests**

| | Elastic modulus (GPa) | Hardness (GPa) |
|---|---|---|
| Natural enamel [a] | 93.89±3.22 | 3.50±0.15 |
| Laser group [a] | 48.29±4.74 | 1.07±0.34 |
| Laser group after A-S remineralization [a] | 60.54±0.83 | 0.97±0.16 |

[a] Data were obtained by the average of 3 indent measurements with the same parameter and expressed as average ±standard deviation.

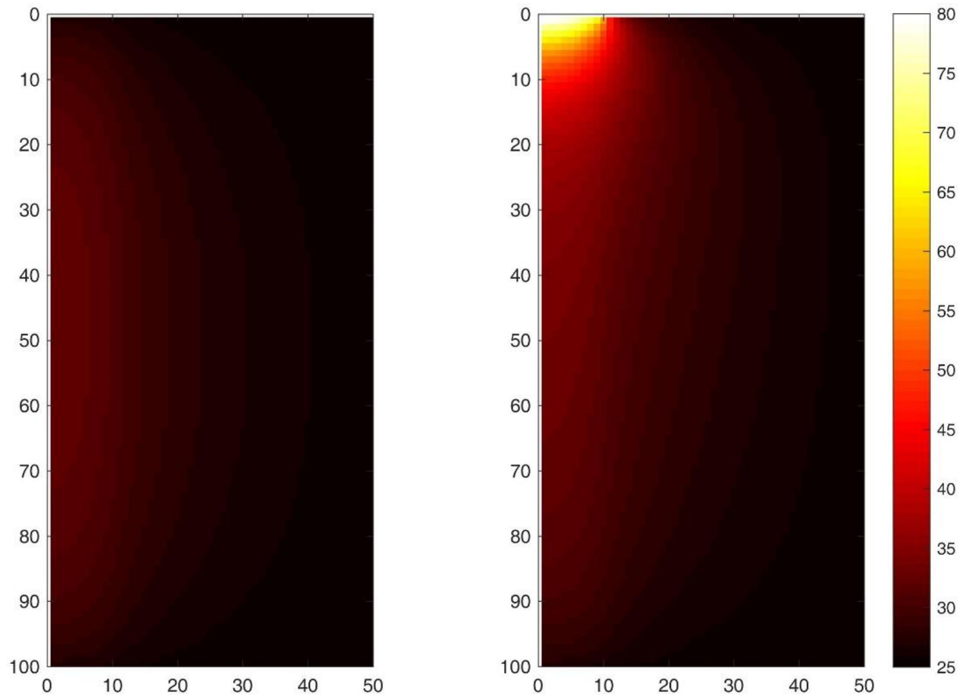


**Fig S4 Simulation results of the temperature distribution of the tooth. Left: tooth without graphite treatment; Right: tooth scraped with graphite.**

## Finite difference method simulation of temperature distribution

The temperature distribution of the tooth after laser treatment is studied through a heat conduction simulation on a simplified tooth model. The tooth is represented by a homogeneous cylinder immersed in an infinite amount of water. The laser is a parallel beam with a uniform circular cross section, and propagate though the axis of the cylinder. The absorption coefficient of the cylinder is uniform and anisotropic, and the scattering of the laser is omitted for simplicity.

Since the model is rotational symmetrical, the heat conduction equations could be formulated in cylinder coordinates. Provided that the density ρ and the thermal capacity c are constant over the temperature range, the equation can be reduced as:

$$\rho c \frac{\partial T}{\partial \tau} = \frac{1}{r}\frac{\partial T}{\partial r} + \frac{\partial^2 T}{\partial r^2} + \frac{\partial^2 T}{\partial z^2} + \frac{q_v}{\lambda}$$

where $T$ is the temperature distribution; $\tau$ is the time; $r$ and $z$ are the length along radial and axial directions, respectively; $q_v$ is the heat generation rate; and $\lambda$ is the heat conductivity.

A uniform cylindrical grid is generated to enable the discretization. However, owing to the symmetry, only a two dimensional slice of the minimum generatrix is studied. The resulted two dimensional uniform grid on this surface has a spatial step length of $d$.

For the boundary grids, they exchange heat with the surrounding stirred water. The temperature of the water $T_{env}$ is kept constant, and the convective heat transfer process is described with Newton's law of cooling.

The internal heat source generates heat at a constant rate. It converts the energy of the absorbed light into heat. The light first interact with the graphite layer of the sample, which absorbs a considerable amount of photons.

Over the total time of simulation $t_{tot}$, the temperature distribution is calculated with the finite-difference method in a central difference scheme approach. Then, the same model without the graphite layer is also simulated using the same method. The major parameters are listed in Table S2. Relative parameters about thermal constants, optical properties and absorption coefficient of graphite are based on reference[1 2 3].

**Table S2 Parameters of the model for simulating surface temperature change by laser**

| Variable | Meaning | Value | Unit |
|---|---|---|---|
| $\rho$ | Density of the tooth | $2.2\times10^{3}$ | kg/m$^3$ |
| $c$ | Specific heat of the tooth | 1255.2 | J/(kg • K) |
| $\lambda$ | Heat conductivity of the tooth | 0.933 | J/(s • m • K) |
| $\mu_a$ | Absorption coefficient of the tooth | 12 | m$^{-1}$ |
| $h$ | Convection heat transfer coefficient | 2000 | J/(s • m$^2$ • K) |
| $\Delta\tau$ | Temporal step length | $5\times10^{-3}$ | s |
| $d$ | Spatial step length | $1\times10^{-4}$ | m |
| $R$ | Radius of the cylinder | $5\times10^{-3}$ | m |
| $H$ | Height of the cylinder | $1\times10^{-2}$ | m |
| $T_{env}$ | Environmental temperature | 25 | ℃ |
| $P_l$ | Power density of the laser | 2 | W |
| $R_l$ | Radius of the laser beam | $1\times10^{-3}$ | m |
| $\mu_a^{\mathrm{G}}$ | Absorption coefficient of graphite | $1\times10^{6}$ | m$^{-1}$ |
| $l^{\mathrm{G}}$ | Thickness of the graphite | $3\times10^{-7}$ | m |

The results of the model with and without graphite coating is shown in Fig. S4. The labels of x- and y-axis are the numbers of grids. The axis of the cylinder is the left edge

of the simulated area.

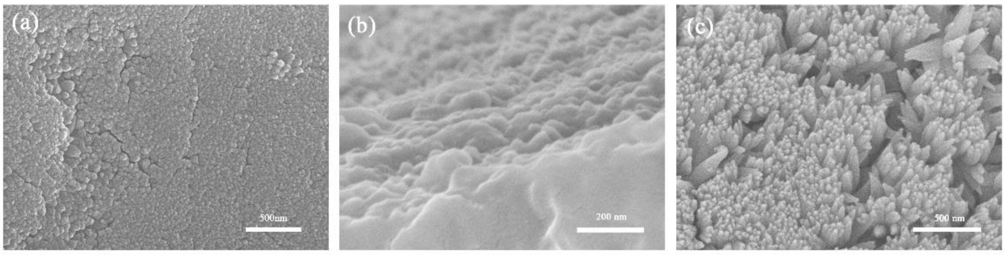


**Figure S5. SEM results of remineralization without graphite / HEDTA. (a), FA was laser-assisted grown on an enamel substrate without graphite. (b), the cross-sectional SEM of (a), the length of the film was hard to identify, which was too thin and just like apatite particles deposited on the enamel. (c), FA was grown on an enamel substrate without HEDTA. Although columnar crystals were grown on enamel, the density of which was low. The orientation of crystals was disordered, which hindered further crystal growth and the formation of a compact crystal film. The mineralization solution used here contained 0.2 M $CaCl_2$, 0.12 M $PO_4^{3-}$ and 0.04 M $F^-$. The laser treatment lasted for 3min, the power of which was 2 W.**

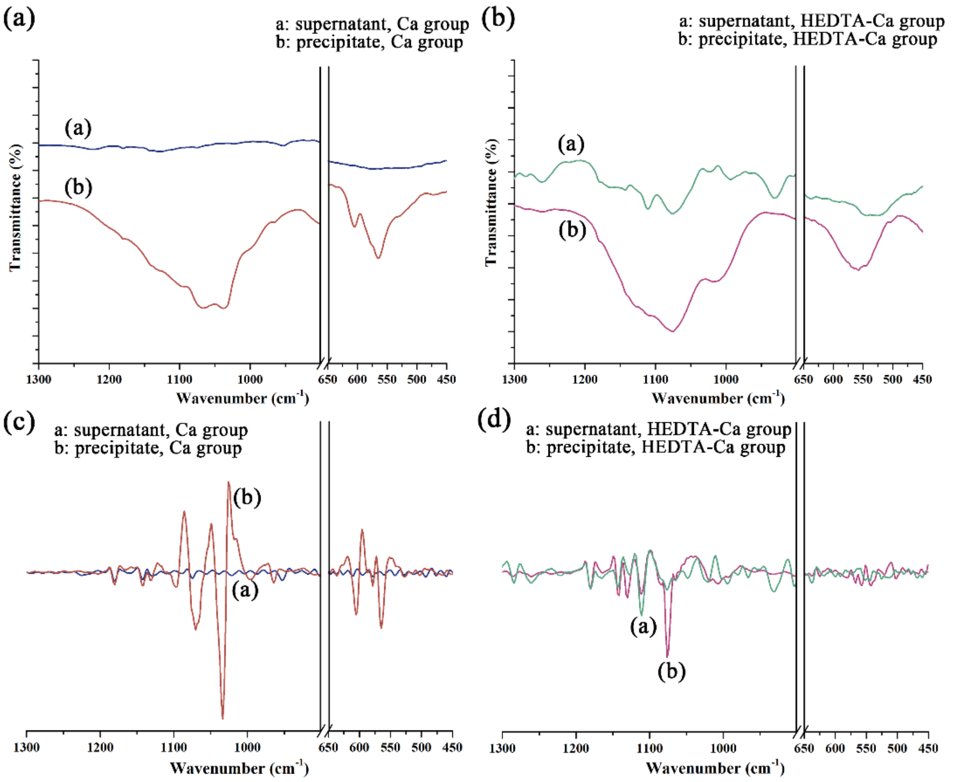


**Figure S6. FT-IR spectra of the precipitate and supernatant. (a)(b), typical FT-IR absorbance spectra of the $\upsilon_1$, $\upsilon_3$ phosphate region of the CaP particles from supernatant and precipitate of different mineralization solution. (c)(d) is the second-derivative spectra of (a)(b).**

FT-IR was used to investigate the effect of chelating agents in our system. For

avoiding the interference of the temperature and the solid interface, neither laser nor substrate was used in this section. Because of the high supersaturation, precipitation occurred at the time when we mixed $Ca^{2+}$ solution with $H_2PO_4^-$ and $F^-$ solution in neutral environment. After centrifugation at 5000 rpm, the precipitate and supernatant were segregated and detected respectively. To further study the spectra and find hidden peaks, second-derivative spectra were calculated and illustrated in Figure S5. Based on the infrared spectra study[4 5] and relative researches, following conclusions can be drawn:

1. When considering the spectrum of precipitate from non-HEDTA group (Fig. S6a), peaks associated with the triply degenerated asymmetric stretching mode of $PO_4^{3-}$ were presented at 1096 $cm^{-1}$ and 1070 $cm^{-1}$. Peaks relative to the triply degenerated bending mode of O-P-O were presented at 603 $cm^{-1}$, 578$cm^{-1}$ and 564 $cm^{-1}$.The symmetric stretching mode of $PO_4^{3-}$ was shown at 960 $cm^{-1}$. However, the presence of peaks at around 990 $cm^{-1}$ confirmed the existence of DCPD. The whole spectrum demonstrates the processing of conversion from ACP to poorly crystalline HA and other acid phosphate phase (DCPD).
2. The spectrum of the supernatant from non-HEDTA group showed few absorption peaks between 1100 $cm^{-1}$ and 900 $cm^{-1}$, implying that nearly all the phosphate groups were consumed (Fig. S6c). In other words, neither PNCs nor ACP existed in the supernatant of the non-HEDTA group.
3. The spectrum of the precipitate from HEDTA group showed a broad and strong peak around 1076 $cm^{-1}$ and a broad peak around 560 $cm^{-1}$, which was in line with the standard spectrum of ACP (Fig. S6b). A weak peak at 1020 $cm^{-1}$ represented the asymmetric stretching mode of $PO_4^{3-}$ from nonstoichiometric apatite. Excluding the effect of absorption peaks from HEDTA, precipitate from HEDTA group was proved to contain plentiful of ACP, which also matched our results from XRD and TEM.
4. Comparing to the spectrum of the precipitate, the one of supernatant from HEDTA group showed pretty strong absorption peaks from HEDTA instead of $PO_4^{3-}$ (Fig. S6b). The strength of $v_3$ region of $PO_4^{3-}$ was nearly equal to that of the peak at 1110 $cm^{-1}$, which belonged to the absorption of C-OH from HEDTA.

Nevertheless, the peak at around 1076 $cm^{-1}$ was still pretty markedly than that from non-HEDTA group (Fig. S6d). The weak peak at 1020 $cm^{-1}$ confirmed the existence of $PO_4^{3-}$ from the nonstoichiometric apatite, which stood for the pre-nucleation clusters. The broad peak around 530 $cm^{-1}$ represented that both $HPO_4^{2-}$ and $PO_4^{3-}$ existed in the supernatant. Considering that in neutral environment $PO_4^{3-}$ was very likely to hydrolyze and form $HPO_4^{2-}$ , the bending mode of which was at 526 $cm^{-1}$ (Fig. S6d), it also proved that other than $HPO_4^{2-}$, PNCs were stabilized in the supernatant.

## Reference


1 Brown, W., Dewey, W. & Jacobs, H. Thermal properties of teeth. *Journal of dental research* **49**, 752-755 (1970).

2 Fried, D., Glena, R. E., Featherstone, J. D. & Seka, W. Nature of light scattering in dental enamel and dentin at visible and near-infrared wavelengths. *Applied optics* **34**, 1278-1285 (1995).

3 Rouleau, F. & Martin, P. Shape and clustering effects on the optical properties of amorphous carbon. *The Astrophysical Journal* **377**, 526-540 (1991).

4 Berzina-Cimdina, L. & Borodajenko, N. *Research of calcium phosphates using Fourier transform infrared spectroscopy*. (INTECH Open Access Publisher, 2012).

5 Gadaleta, S., Paschalis, E., Betts, F., Mendelsohn, R. & Boskey, A. Fourier transform infrared spectroscopy of the solution-mediated conversion of amorphous calcium phosphate to hydroxyapatite: new correlations between X-ray diffraction and infrared data. *Calcified tissue international* **58**, 9-16 (1996).
